# Echogenic Technology Improves Cannula Visibility during Ultrasound-Guided Internal Jugular Vein Catheterization via a Transverse Approach

**DOI:** 10.1155/2012/306182

**Published:** 2012-05-10

**Authors:** Konstantinos Stefanidis, Nicos Pentilas, Stavros Dimopoulos, Serafim Nanas, Richard H. Savel, Ariel L. Shiloh, John Poularas, Michel Slama, Dimitrios Karakitsos

**Affiliations:** ^1^Radiology Department, Evangelismos University Hospital, Athens, Greece; ^2^Intensive Care Unit, General State Hospital of Athens, Athens, Greece; ^3^1st Critical Care Department, Evangelismos University Hospital, Athens, Greece; ^4^Jay B. Langner Critical Care Service, Department of Medicine, Montefiore Medical Center, Albert Einstein College of Medicine, NY, USA; ^5^Intensive Care Unit, CHU Sud, 80054 Amiens Cedex 1, France, France; ^6^Unité INSERM 1088, University Picardie Jules Vernes, Amiens, France

## Abstract

*Objective.* Echogenic technology has recently enhanced the ability of cannulas to be visualized during ultrasound-guided vascular access. We studied whether the use of an EC could improve visualization if compared with a nonechogenic vascular cannula (NEC) during real-time ultrasound-guided internal jugular vein (IJV) cannulation in the intensive care unit (ICU). *Material and Methods.* We prospectively enrolled 80 mechanically ventilated patients who required central venous access in a randomized study that was conducted in two medical-surgical ICUs. Forty patients underwent EC and 40 patients were randomized to NEC. The procedure was ultrasound-guided IJV cannulation via a transverse approach. *Results.* The EC group exhibited increased visibility as compared to the NEC group (88%  ± 8% versus 20%  ± 15%, resp. *P* < 0.01). There was strong agreement between the procedure operators and independent observers (*k* = 0.9; 95% confidence intervals assessed by bootstrap analysis = 0.87–0.95; *P* < 0.01). Access time (5.2 s ± 2.5 versus 10.6 s ± 5.7) and mechanical complications were both decreased in the EC group compared to the NEC group (*P* < 0.05). *Conclusion.* Echogenic technology significantly improved cannula visibility and decreased access time and mechanical complications during real-time ultrasound-guided IJV cannulation via a transverse approach.

## 1. Introduction

Real-time ultrasound-guided central venous cannulation has been associated with higher success rates, faster access times, and a reduction in mechanical complications when compared to landmark techniques, especially for the cannulation of the internal jugular vein (IJV) [1–6]. The ultrasound-guided method via a longitudinal approach has been favored since it offered another view to visualize the needle tip in the lumen and the back wall of the vein [6]. However, the transverse axis approach has been the standard monoplanar ultrasound view since the introduction of the above technique [7], but it was rather problematic in visualizing the cannula and thus controlling its depth without arterial puncture or transfixion of the vein [8, 9]. This may be particularly relevant to the intensive care unit (ICU) setting as the clarity of two-dimensional (2D) ultrasound images is oftentimes affected in critical care patients by the presence of various factors such as obesity, subcutaneous air and/or edema, trauma and mechanical ventilation, while complications may occur even under ultrasound guidance [6–12]. Cannula visualization is fundamental to the safety and efficacy of all ultrasound-guided methods, but no single technology meant to improve cannula echogenicity has been widely adopted or studied in the ICU setting [13–20]. Recently, a vascular cannula (VascularSono, Pajunk, GmbH, Medizintechnologie, Geisingen, Germany) incorporating “Cornerstone” reflectors on the distal 2 cm, to increase echogenicity, was developed based on technology previously used in regional anesthesia cannulas [16]. We hypothesized that the use of an echogenic vascular cannula (EC) would improve visualization when compared with a nonechogenic vascular cannula (NEC) (Arrow Howes, PA, USA) during real-time ultrasound-guided internal jugular vein (IJV) cannulation via a transverse approach.

## 2. Materials and Methods

During 2011, eighty patients who required central venous access were prospectively enrolled in this randomized study that was conducted in two medical-surgical ICUs. Forty patients underwent EC and 40 patients were randomized to NEC. The procedure was ultrasound-guided IJV cannulation via a transverse approach. All patients were sedated and mechanically ventilated. Randomization was performed by means of a computer-generated random-number table and patients were stratified with regard to age, gender, and body mass index (BMI). Block randomization was used to ensure equal numbers of patients in the above groups [6]. All physicians who performed the procedures had at least five years of experience in central venous catheter placement. The study was approved by the Institutional Ethics Committee, and appropriate informed consent was obtained. Chest radiography was used to assess catheter placement after the procedure. Mechanical complications were defined as arterial puncture, hematoma, hemothorax, pneumothorax, and catheter misplacement [6].

### 2.1. Real-Time Ultrasound-Guided IJV Cannulation

All patients were placed in Trendelenburg position and were cannulated as described in detail by Karakitsos et al. [6]. Triple-lumen catheters were used in all cases and all procedures were performed under controlled, nonemergent conditions in the ICU. Standard sterile precautions were utilized. The EC and NEC were both 18-gauge cannulas specifically intended for use in vascular access. Ultrasonography was performed with an HD11 XE ultrasound machine (Philips, Andover, MA, USA) equipped with a high-resolution 7.5–12 MHz transducer, which was covered with sterile ultrasonic gel and wrapped in a sterile sheath (Microtec medical intraoperative probe cover, 12 cm × 244 cm). Vessels were cannulated using the Seldinger technique under real-time ultrasound guidance.

### 2.2. Data Acquisition, Study Protocol, and Outcome Measures

The cannulation was performed by a single operator and was observed by a second physician. The operators and observers were blinded to the cannula used. Following each procedure, the operator and the observer were asked to score the percentage of time they were able to continuously visualize the cannula; a 10-point scale was used (ranging from 1 = 0%–10%, to 10 = 90%–100%). The observer measured access time, number of attempts, and complications. Access time was defined as the time between penetration of skin and aspiration of venous blood. Data was collected using a standardized form and was entered in a database. We documented baseline patient characteristics, side of catheterization, the presence of risk factors for difficult venous cannulation, previous difficulties during cannulation, previous mechanical complications, known vascular abnormalities, and untreated coagulopathy (international normalization ratio > 2; activated partial thromboplastin time >1.5; platelets <50 × 109  litre − 1) [6].

## 3. Statistical Analysis

Data were expressed as mean ± standard deviation (SD). The Student's *t-*test for independent mean, *χ* ^2^ analysis, or Fisher's exact test where appropriate were used to identify differences between the two groups. A *P* value (two-sided in all tests) of <0.05 was considered significant. Study power was based on data from a previous cannula visibility study, which were adjusted for our intervention [19]. Assuming data to be nonparametric, power sample analysis gave a minimum sample size of 40 cannulations. Wilcoxon rank sum test was used to compare cannula visibility data for the 2 groups. The agreement between the operator and observer cannula visibility results was evaluated by Cohen's weighted kappa, while 2.5th, and 97.5th percentiles of 5,000 bootstrap replicates estimated 95% confidence intervals. The bootstrap is a resampling method used for estimating a distribution, from which various measures of interest can be calculated [21]. Statistical analysis was performed using SPSS, version 11.0 (SPSS Inc. Chicago, IL, USA).

## 4. Results

 Baseline characteristics of the study population are presented in Table 1. There were no significant differences in age, gender, body mass index (BMI), and presence of risk factors for difficult venous cannulation between the NEC and the EC groups; moreover no cases of preexisting thrombosis were identified.

**Figure 1:**
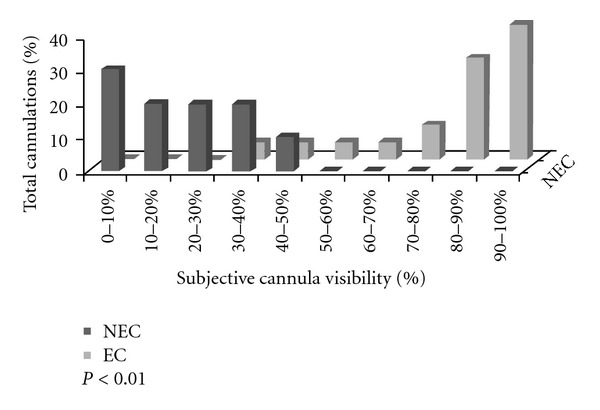
Subjective cannula visibility assessments (echogenic cannula, EC: gray; nonechogenic cannula, NEC: black).

**Figure 2:**
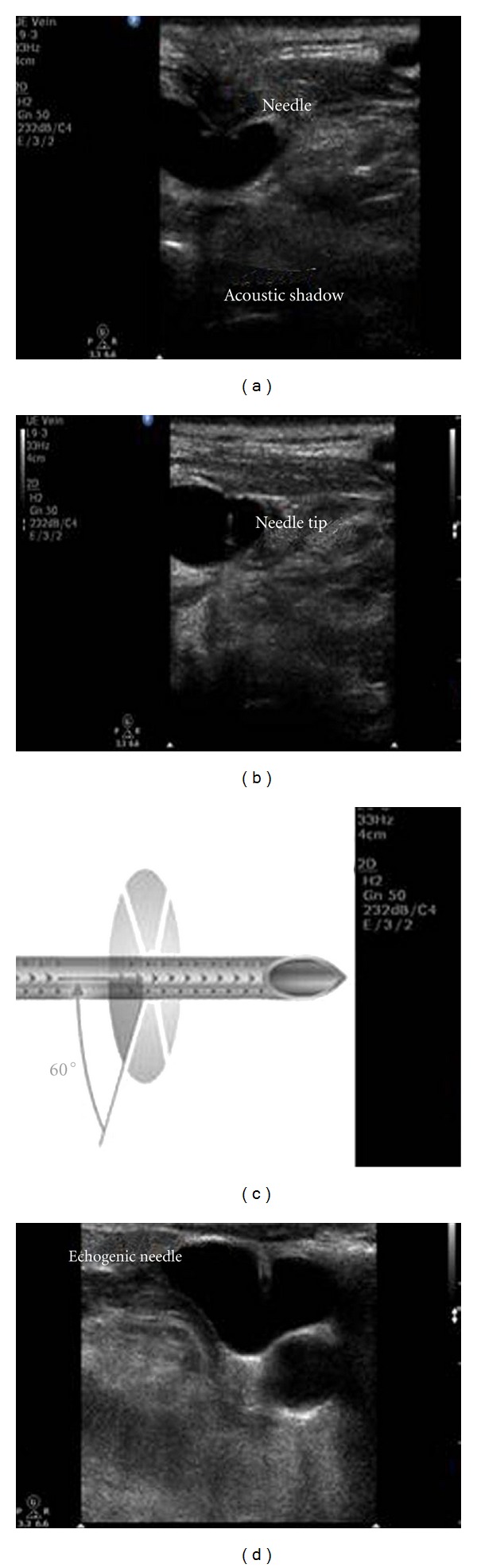
Nonechogenic cannula entering the anterior wall (a) and depicted within the lumen of the internal jugular vein, on the transverse axis (b); please observe that the echogenic cannula incorporates “cornerstone” reflectors arranged at its distal 2 cm (c), which increases dramatically its visibility (d).

 Results of cannula visibility are presented on Figure 1. Operators reported improved cannula visualization in the EC group when compared to the NEC group (88% ± 8% versus 20% ± 15%, resp.; *P* < 0.01). Also, operators reported that when using the NEC via the transverse approach they might have visualized it, but surely have noticed its acoustic shadow (Figure 2). In contrast, the echogenic vascular cannula could be clearly identified, even if the insonation angle was slightly modified (Figure 2). Finally, the agreement between the operators and observers was statistically significant (kappa = 0.9; 95% confidence intervals assessed by bootstrap analysis = 0.87–0.95; *P* < 0.01). 

Results of the secondary outcomes are presented in Table 2. Access time (5.2 s ± 2.5 versus 10.6 s ± 5.7) and mechanical complications, notably hematomas (0% versus 10%), were both decreased in the EC group compared to the NEC group (*P* < 0.05).

## 5. Discussion 

Our study demonstrated improved cannula visibility with the use of EC during ultrasound-guided IJV cannulation via a transverse approach. The latter has been the standard monoplanar 2D view since the introduction of the ultrasound technique [7]. Intrinsically, cannulation of a vessel using the transverse approach often limits cannula visibility; hence controlling the trajectory of the cannula may be problematic, especially when the 2D image is of low quality due to various factors (i.e., subcutaneous air and/or edema, trauma, etc.) [1–12].

 Nevertheless, we found that the use of EC statistically increased the likelihood of continued successful cannula visualization. This could be attributed to the fact that the EC is brightly echogenic as it incorporates “Cornerstone” reflectors mainly arranged at the distal 2 cm of the needle. These reflectors guarantee the visibility of the cannula shaft, independent of the puncture angle according to the manufacturer. The principle is the same as that used in bicycle reflectors, where light is reflected back to its source regardless of the angle at which it approaches [16–19]. The present results demonstrated that using the EC resulted in significantly reduced access times and mechanical complications. Notably, once IJV cannulation is pursued via a transverse approach, the adjacent carotid artery may be a potential “target” for the cannula, especially if the operator cannot fully control its trajectory [3–9].

The present methodology was designed to test EC in actual clinical practice in the ICU, where image acquisition is affected or limited by the presence of various factors such as obesity, subcutaneous air, edema, trauma, and mechanical ventilation [1–12]. The use of EC may improve image acquisition and success rates in technically challenging cases of vascular access. Moreover, we should underline that the transverse approach is less technically demanding compared to the longitudinal one under ultrasound guidance, and thus can be easily applied by inexperienced operators [1–12]. This technical “advantage” can be further enhanced by the implementation of echogenic cannulas, and hence resulting in a simpler ultrasound-guided method but with optimal cannula visibility results. There is no definitive method for objective assessment of cannula visibility. Previous studies used scoring systems with skilled observers rating static images [14–19]. We aimed to examine cannula visibility during IJV cannulation, under real-time clinical conditions. Although interpretation of dynamic 2D ultrasound images remains subjective, we used an analytical 10-point scale along with a dual evaluation model of operators and observers. We demonstrated that high operator and observer agreement existed between the subjective estimations of cannula visibility rates. The study has several limitations. Despite the fact that the operators were blinded at the initiation of the procedure, the two vascular cannulas inherently exhibited different ultrasonographic appearance, and could possibly be differentiated. Although we demonstrated a significant reduction in hematoma formation, we failed to find a significant reduction of major mechanical complications. This may be due to the fact that our baseline mechanical complication rate was extremely low (given the fact that our study group is highly skilled in ultrasound-guided vascular access) and that the sample size was rather small. Concluding, the present investigation demonstrated that echogenic technology significantly improved cannula visibility and decreased access time during real-time ultrasound-guided IJV cannulation via a transverse approach. Our data provide clinical rationale to study the evolving field of enhanced echogenic ultrasound technology. Further studies are required to determine if EC is cost-effective and changes overall outcomes in the ICU.

## Figures and Tables

**Table 1:** Baseline characteristics of the study population; values are presented either in percentages or as mean ± SD.

Characteristics	EC group (*n* = 40)	NEC group (*n* = 40)
Age (years)	45 ± 9.5	46 ± 10.9
Gender (male/female ratio)	0.49 ± 0.4	0.5 ± 0.5
APACHE II score	20.6 ± 2.1	20.8 ± 2.4
Diagnosis upon admission		
Trauma without brain injury	15 (37.5%)	15 (37.5%)
Trauma with brain injury	15 (37.5%)	15 (37.5%)
Burn	1 (2.5%)	0 (0%)
ARDS	2 (5%)	2 (5%)
Sepsis	5 (12.5%)	7 (17.5%)
Postsurgical complications	2 (5%)	1 (2.5%)
Side of catheterization (left/right)	14/26	12/28
Body mass index (kg/m^2^)	21.1 ± 3.6	21.8 ± 3.9
Prior catheterization	7 (17.5%)	5 (12.5%)
Limited sites for access attempts	5 (12.5%)	5 (12.5%)
Previous difficulties during Catheterization	9 (22.5%)	7 (17.5%)
Previous mechanical complications	6 (15%)	4 (10%)
Known vascular abnormality	1 (2.5%)	1 (2.5%)
Untreated coagulopathy	1 (2.5%)	1 (2.5%)
Skeletal deformity	1 (2.5%)	1 (2.5%)

APACHE II score: acute physiology and chronic health evaluation score II; ARDS: acute respiratory distress syndrome; NEC: nonechogenic cannula, EC: echogenic cannula.

**Table 2:** Secondary outcome measures in the EC group versus the NEC group.

Outcome measures	EC group (*n* = 40)	NEC group (*n* = 40)
Access time (sec)	5.2 ± 2.5 (4.5–12.4)*	10.6 ± 5.7 (8.1–17.3)
Success rate (%)	40 (100%)	40 (100%)
Average number of attempts	1 ± 0.2 (1–1.3)	1.1 ± 0.4 (1–1.7)
Artery puncture	0 (0%)	1 (2.5%)
Hematoma	0 (0%)*	4 (10%)
Pneumothorax	0 (0%)	0 (0%)
Hemothorax	0 (0%)	0 (0%)

EC: echogenic cannula, NEC: nonechogenic cannula; Comparisons between the NEC and the EC group of patients; *P* < 0.05*; Access time and average number of attempts are expressed as mean ± SD (95% confidence intervals).

## References

[1] Randolph AG, Cook DJ, Gonzales CA, Pribble CG (1996). Ultrasound guidance for placement of central venous catheters: a meta- analysis of the literature. *Critical Care Medicine*.

[2] Machi J, Takeda J, Kakegawa T (1987). Safe jugular and subclavian venipuncture under ultrasonographic guidance. *American Journal of Surgery*.

[3] Bond DM, Champion LK, Nolan R (1989). Real-time ultrasound imaging aids jugular venipuncture. *Anesthesia and Analgesia*.

[4] Mallory DL, McGee WT, Shawker TH (1990). Ultrasound guidance improves the success rate of internal jugular vein cannulation. a prospective, randomized trial. *Chest*.

[5] Denys BG, Uretsky BF, Reddy PS (1993). Ultrasound-assisted cannulation of the internal jugular vein: a prospective comparison to the external landmark-guided technique. *Circulation*.

[6] Karakitsos D, Labropoulos N, De Groot E (2006). Real-time ultrasound-guided catheterisation of the internal jugular vein: a prospective comparison with the landmark technique in critical care patients. *Critical Care*.

[7] Legler D, Nugent M (1984). Doppler localization of the internal jugular vein facilitates central venous cannulation. *Anesthesiology*.

[8] Hayashi H, Amano M (2002). Does ultrasound imaging before puncture facilitate internal jugular vein cannulation? prospective randomized comparison with landmark-guided puncture in ventilated patients. *Journal of Cardiothoracic and Vascular Anesthesia*.

[9] Augoustides JG, Diaz D, Weiner J, Clarke C, Jobes DR (2002). Current practice of internal jugular venous cannulation in a university anesthesia department: influence of operator experience on success of cannulation and arterial injury. *Journal of Cardiothoracic and Vascular Anesthesia*.

[10] Sznajder JI, Zveibil FR, Bitterman H (1986). Central vein catheterization. failure and complication rates by three percutaneous approaches. *Archives of Internal Medicine*.

[11] Mansfield PF, Hohn DC, Fornage BD, Gregurich MA, Ota DM (1994). Complications and failures of subclavian-vein catheterization. *New England Journal of Medicine*.

[12] Blaivas M, Adhikari S (2009). An unseen danger: frequency of posterior vessel wall penetration by needles during attempts to place internal jugular vein central catheters using ultrasound guidance. *Critical Care Medicine*.

[13] Maecken T, Zenz M, Grau T (2007). Ultrasound characteristics of needles for regional anesthesia. *Regional Anesthesia and Pain Medicine*.

[14] Edgcombe H, Hocking G (2010). Sonographic identification of cannula by specialists and novices: a blinded comparison of 5 regional block cannulas in fresh human cadavers. *Regional Anesthesia and Pain Medicine*.

[15] Schafhalter-Zoppoth I, McCulloch CE, Gray AT (2004). Ultrasound visibility of needles used for regional nerve block: an in vitro study. *Regional Anesthesia and Pain Medicine*.

[16] Deam RK, Kluger R, Barrington MJ, McCutcheon CA (2007). Investigation of a new echogenic needle for use with ultrasound peripheral nerve blocks. *Anaesthesia and Intensive Care*.

[17] Bondestam S, Kreula J (1989). Cannula echogenicity. A study with real time ultrasound. *Invest Radiol*.

[18] Nichols K, Wright LB, Spencer T, Culp WC (2003). Changes in ultrasonographic echogenicity and visibility of cannulas with changes in angles of insonation. *Journal of Vascular and Interventional Radiology*.

[19] Hebard S, Hocking G ( 2011). Echogenic technology can improve cannula visibility during ultrasound-guided regional anesthesia. *Regional Anesthesia and Pain Medicine*.

[20] Sprotte G, Schedel R, Pajunk H, Pajunk H (1987). An 'atraumatic' universal cannula for single-shot regional anesthesia: clinical results and a 6 year trial in over 30,000 regional anesthesias. *Regional Anesthesia*.

[21] Efron B, Tibshirani RJ (1993). *An introduction to the Bootstrap*.

